# The bioenergetic “CK Clamp” technique detects substrate-specific changes in mitochondrial respiration and membrane potential during early VML injury pathology

**DOI:** 10.3389/fphys.2023.1178213

**Published:** 2023-04-04

**Authors:** Jennifer McFaline-Figueroa, Edward T. Hunda, Junwon Heo, Elizabeth A. Winders, Sarah M. Greising, Jarrod A. Call

**Affiliations:** ^1^ Department of Physiology and Pharmacology, University of Georgia, Athens, GA, United States; ^2^ Regenerative Biosciences Center, University of Georgia, Athens, GA, United States; ^3^ School of Kinesiology, University of Minnesota, Minneapolis, MN, United States

**Keywords:** muscle metabolism, muscle bioenergetics, muscle injury, oroboros oxygraph 2K, HORIBA

## Abstract

Volumetric muscle loss (VML) injuries are characterized by non-recoverable loss of tissue resulting in contractile and metabolic dysfunction. The characterization of metabolic dysfunction in volumetric muscle loss-injured muscle has been interpreted from permeabilized myofiber respiration experiments involving saturating ADP levels and non-physiologic ATP:ADP concentration ratios. The extent to which this testing condition obscures the analysis of mitochondrial (dys) function after volumetric muscle loss injury is unclear. An alternative approach is described that leverages the enzymatic reaction of creatine kinase and phosphocreatine to assess mitochondrial respiration and membrane potential at clamped physiologic ATP:ADP ratios, “CK Clamp.” The objective of this study was to validate the CK Clamp in volumetric muscle loss-injured muscle and to detect differences that may exist between volumetric muscle loss-injured and uninjured muscles at 1, 3, 5, 7, 10, and 14 days post-injury. Volumetric muscle loss-injured muscle maintains bioenergetic features of the CK Clamp approach, i.e., mitochondrial respiration rate (JO_2_) titters down and mitochondrial membrane potential is more polarized with increasing ATP:ADP ratios. Pyruvate/malate/succinate-supported JO_2_ was significantly less in volumetric muscle loss-injured muscle at all timepoints compared to uninjured controls (−26% to −84%, *p* < 0.001) and electron conductance was less at day 1 (−60%), 5 (−52%), 7 (−35%), 10 (−59%), and 14 (−41%) (*p* < 0.001). Palmitoyl-carnitine/malate-supported JO_2_ and electron conductance were less affected following volumetric muscle loss injury. volumetric muscle loss-injury also corresponded with a more polarized mitochondrial membrane potential across the clamped ATP:ADP ratios at day 1 and 10 (pyruvate and palmitoyl-carnitine, respectively) (+5%, *p* < 0.001). This study supports previous characterizations of metabolic dysfunction and validates the CK Clamp as a tool to investigate bioenergetics in traumatically-injured muscle.

## Highlights

• This study validates the CK Clamp technique, an approach to assess mitochondrial respiration and membrane potential at physiological ATP:ADP concentration ratios, as a tool to investigate mitochondrial bioenergetics in VML-injured muscle
• This study demonstrates a relatively greater deficit in carbohydrate-supported metabolism compared to fat-supported metabolism during early VML pathology
• The findings from this study utilizing the CK Clamp technique support previous reports of VML-injured muscle metabolic dysfunction that involved supra-physiological ATP:ADP ratio experimental conditions

## Introduction

Volumetric muscle loss (VML) injuries encompass a group of pathologies characterized by the frank loss of muscle tissue due to trauma or surgical removal (Grogan et al., 2011). The result of these injuries is the marked, sustained decline of muscle strength (Grogan et al., 2011; Corona et al., 2015; Greising et al., 2017), mobility, and greater fibrotic tissue deposition (Corona et al., 2015; Garg et al., 2015; Chao et al., 2019; Southern et al., 2019; Dalske et al., 2021). VML pathology is also associated with changes in skeletal muscle and whole-body metabolism. For example, following VML injury, there is less mitochondrial respiration in permeabilized myofibers (Greising et al., 2018; Southern et al., 2019; McFaline-Figueroa et al., 2022), less mitochondrial enzyme activities (Aurora et al., 2014), less activation of *PGC1α* (genetic marker of mitochondrial biogenesis) (Aurora et al., 2014; Southern et al., 2019), and changes in whole-body diurnal metabolism, resulting in lower oxidation of carbohydrates (Dalske et al., 2021; Raymond-Pope et al., 2022).

A particular limitation of the existing mitochondrial respiration studies in the context of VML-injured myofibers is that the experiments were conducted under non-physiological testing conditions (i.e., supra-physiological levels of ADP) that may exaggerate the findings and interpretations. To directly address this limitation, the current study was designed to validate a physiological stress test (Glancy et al., 2008; Fisher-Wellman et al., 2018) that assesses mitochondrial oxygen consumption and mitochondria membrane potential at specific ATP re-synthesis demand states in the context of a VML injury. To determine if fuel metabolism at the tissue-level mirrored whole-body changes in carbohydrate and fat oxidations after VML injury (Dalske et al., 2021), VML-injured myofibers ability to utilize carbohydrates and fat substrates during the physiological stress test was assessed. This study focused on the first 2 weeks of pathology following VML injury when there are documented changes to the mitochondrial network (Southern et al., 2019).

## Materials and methods

### Animals

Male C57BL/6 mice (n = 42) were housed at 20°C–23°C on a 12:12-h light-dark cycle, with food and water provided *ad libitum*. At the time of randomization to experimental groups, all mice were between 11 and 12 weeks of age. All procedures were approved and performed in accordance with the guidelines and regulations of the Institutional Animal Care and Use Committee at the University of Georgia.

### Experimental design

VML-injured mice underwent unilateral VML injury and were sacrificed at the following timepoints post-injury (n = 6–8/group): 1, 3, 5, 7, 10, and 14-days. At the study endpoint, muscle mass, carbohydrate and fat-supported oxygen consumption and mitochondrial membrane potential were assessed. Sample size was determined based on the coefficient of variance for permeabilized muscle fiber respiration (∼15%), a capacity to detect at minimum 15% changes across groups, and a Power of 0.8 (calculated *a priori* in JMP^®^, Version 16.0. SAS Institute Inc., Cary, NC).

### Volumetric muscle loss injury

Unilateral VML injury to the left hindlimb plantar flexors (gastrocnemius, soleus, plantaris) provided volumetric removal of muscle tissue from anesthetized (isoflurane 1.5%–3.0%) mice, as previously described (Greising et al., 2018; Southern et al., 2019). Mice were administered buprenorphine (1.2 mg/kg; s. q.) prior to a single incision being made in the mid-gastrocnemius from distal to proximal exposing posterior compartment muscles. Using a 4 mm standardized biopsy punch of the posterior muscles resulted in a removal of ∼15% (27.2 ± 1.2 mg) of muscle tissue. Any bleeding was stopped with light pressure. Skin incisions were closed with 6.0 silk sutures and mice were monitored through recovery.

### CK Clamp mitochondrial respiration

Permeabilized myofiber high-resolution respirometry was performed using an Oroboros Oxygraph-2K (Oroboros Instruments, Innsburck, Austria). Experiments were performed at 30°C in Buffer Z supplemented with ATP (5 mM), creatine (5 mM), phosphocreatine (PCr, 1 mM) and creatine kinase (CK, 20 U/mL). Higher experimental temperatures (e.g., 37°C) are associated with greater respiration rates than can deplete chamber oxygen levels. Re-oxygenation of chambers can be monitored within the Oxygraph-2K chambers, but not in the device used for parallel mitochondrial membrane potential detail below. Therefore, 30°C was selected to maintain an appropriate oxygen concentration for the entirety of the experiment and be consistent between testing platforms. For each experiment, permeabilized skeletal myofiber bundles (∼2 mg) from the gastrocnemius muscles were energized with carbohydrates fuels (4 mM Pyruvate, 1 mM Malate, 20 mM Succinate) or fat fuels (4 mM Palmitoyl-carnitine, 1 mM Malate) and 5 mM ATP to energize the mitochondria. Mitochondrial oxygen consumption was determined using a CK Clamp technique where steady-state oxygen consumption rates are measured while manipulating the free energy of ATP hydrolysis (∆G_ATP_), calculated through an online resource (https://dmpio.github.io/bioenergetic-calculators/ck_clamp/), as previously described (Fisher-Wellman et al., 2018). Sequential PCr titrations were made to final concentrations of 1, 2, 4, 7, 16, 31 mM. All high-resolution respirometry steady-state oxygen consumption data (JO_2_) were corrected for basal oxygen consumption and then normalized by citrate synthase (CS) activity, an indirect measure/estimate of mitochondrial content (Larsen et al., 2012).

### Mitochondria membrane potential

Fluorescence determination of mitochondrial membrane potential was performed using permeabilized myofibers (∼2 mg) in parallel with high-resolution respirometry using a Horiba Spectrofluorometer (FluoroMax Plus-C; Horiba Instruments Inc., Atlanta, GE, United States) as previously described (Scaduto and Grotyohann, 1999; Fisher-Wellman et al., 2018). The membrane potential was determined *via* tetramethylrhodamine methyl ester (TMRM) at 30°C, which parallelly matched the protocol with the CK clamp assay with constant stirring. TMRM excitation/emission [(572/590 nm)/(551/590 nM)] fluorescence is quenched (i.e., 572/551 ratio increases) with greater mitochondrial membrane polarization. The 572/551 ratio is reported herein because there is currently no report of the 572/551 ratio conversion to millivolts validated in permeabilized muscle fiber bundles to our knowledge.

### Citrate synthase activity

Mitochondrial content was analyzed by CS activity as previously described (Southern et al., 2019) using remaining muscle fiber collections for oxygen consumption experiments.

### Statistical analysis

All data is represented as mean ± standard deviation. A one-way ANOVA was used to determine differences between uninjured controls and VML-injured myofibers across timepoints. Uninjured, contralateral control limbs from animals at all timepoints were not statistically different (one-way ANOVA, *p* ≥ 0.1078) and were, therefore, pooled together for analysis. All statistical analyses were performed using JMP statistical software, all graphs were constructed using Prism (GraphPad Prism version 9.4.1 for Windows, GraphPad Software, San Diego, California United States).

## Results and discussion

In agreement with previous reports (Greising et al., 2018; McFaline-Figueroa et al., 2022), the posterior VML injury model resulted in a non-recoverable loss of muscle mass, even when accounting for differences in body mass (Table 1). Although contractile function was not assessed in this study, this model is associated with a ∼80% strength deficit at 3- and 7-days post-injury (Southern et al., 2019) that is largely sustained at 2-month post-injury [−36%, (McFaline-Figueroa et al., 2022)] compared to uninjured, age-matched controls.

**TABLE 1:** Mouse body mass and gastrocnemius muscle mass.

	Experimental group							
	1-day	3-day	5-day	7-day	10-day	14-day	Combined Control	OWA *p*-value
Body Mass (g)	26.9 ± 1.4	28.5 ± 1.0	28.5 ± 0.9	29.2 ± 0.1	31.0 ± 1.6	31.8 ± 0.4	29.3 ± 1.0	-
Gastrocnemius Mass (mg)	126.0 ± 8.5*	130.3 ± 9.6*	157.5 ± 9.4*	138.9 ± 7.3*	133.7 ± 8.4*	134.3 ± 4.7*	174.3 ± 16.0	<0.0001
Gastrocnemius Mass/Body Mass Ratio (mg/g)	4.7 ± 0.2*	4.6 ± 0.5*	5.4 ± 0.3	4.7 ± 0.2*	4.3 ± 0.1*	4.2 ± 0.2*	5.5 ± 0.5	<0.0001

Values are means ± SD; * significantly different than Control. One-Way ANOVA, Tukey HSD, post-hoc test. Each group reflects n = 6.

### Rationale for CK Clamp respirometry approach

**FIGURE 1:**
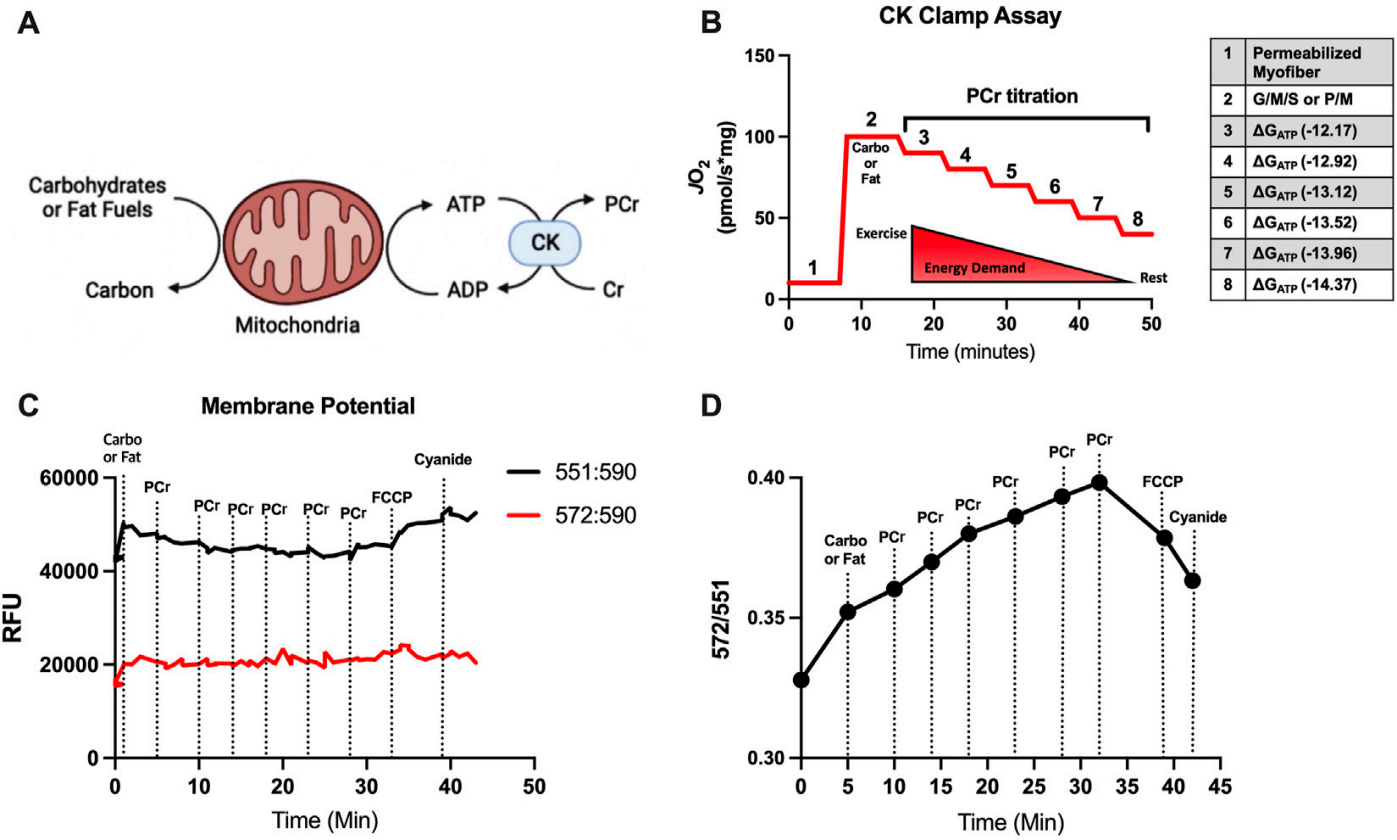
Conceptual framework for CK Clamp technique and mitochondrial membrane potential evaluation. **(A)** Creatine kinase (CK) is catalyzes the reversible reaction of creatine (Cr) and ATP to produce phosphocreatine (PCr) and ADP. **(B)** During the CK Clamp test, the ATP re-synthesis demand (∆G_ATP_) can be manipulated by titrating PCr and has the effect of decreasing oxygen consumption (JO_2_). **(C, D)** A representative mitochondrial membrane test detecting the change in TMRM throughout the CK Clamp. The ratio of the 572-to-551 excitation/emission fluorescence is used to indirectly assess the polarization of the mitochondrial membrane. The reversing of mitochondrial membrane polarization was confirmed by sequential addition of an uncoupler (FCCP) and cyanide.

Standardized mitochondrial oxygen consumption tests at supra-physiological concentrations of ADP can lead to misinterpretations of mitochondrial respiration because mitochondria rarely function *in vivo* at these extremely low ATP/ADP ratios (Schmidt et al., 2021). Furthermore, mitochondrial energy transduction involves a network of dehydrogenases influencing cellular redox status [e.g., NAD(P)H/NAD(P)], electron transport chain proton pumping and establishment of the proton motive force, and the dissipation of this proton motive force during ATP synthesis based on energy demand (i.e., ATP/ADP ratio) (Fisher-Wellman et al., 2018; Schmidt et al., 2021). A multi-dimensional diagnostic assessment is therefore appropriate to better assess mitochondrial function under physiological ranges of ATP/ADP ratios. The concept described by Glancy et al. (Glancy et al., 2013), then later optimized by Fisher-Wellman et al. (Fisher-Wellman et al., 2018; McLaughlin et al., 2020) and employed here, uses the enzymatic reaction of CK and PCr to stepwise “clamp” ATP/ADP ratios a different ATP free energies such that a high demand for ATP re-synthesis is −12.17 ∆G_ATP_ and a low demand for ATP re-synthesis is −14.37 ∆G_ATP_ (Figures 1A, B). When ADP phosphorylation (i.e., ATP re-synthesis) is strongly coupled to respiration (i.e., oxygen consumption), then JO_2_ is expected to be titrated down proportionately to a rise in ∆G_ATP_. Herein, we simultaneously assessed JO2 and the proton motive force, or mitochondrial membrane potential (Figures 1C, D).

### Validation of CK Clamp in VML-Injured myofibers

**FIGURE 2:**
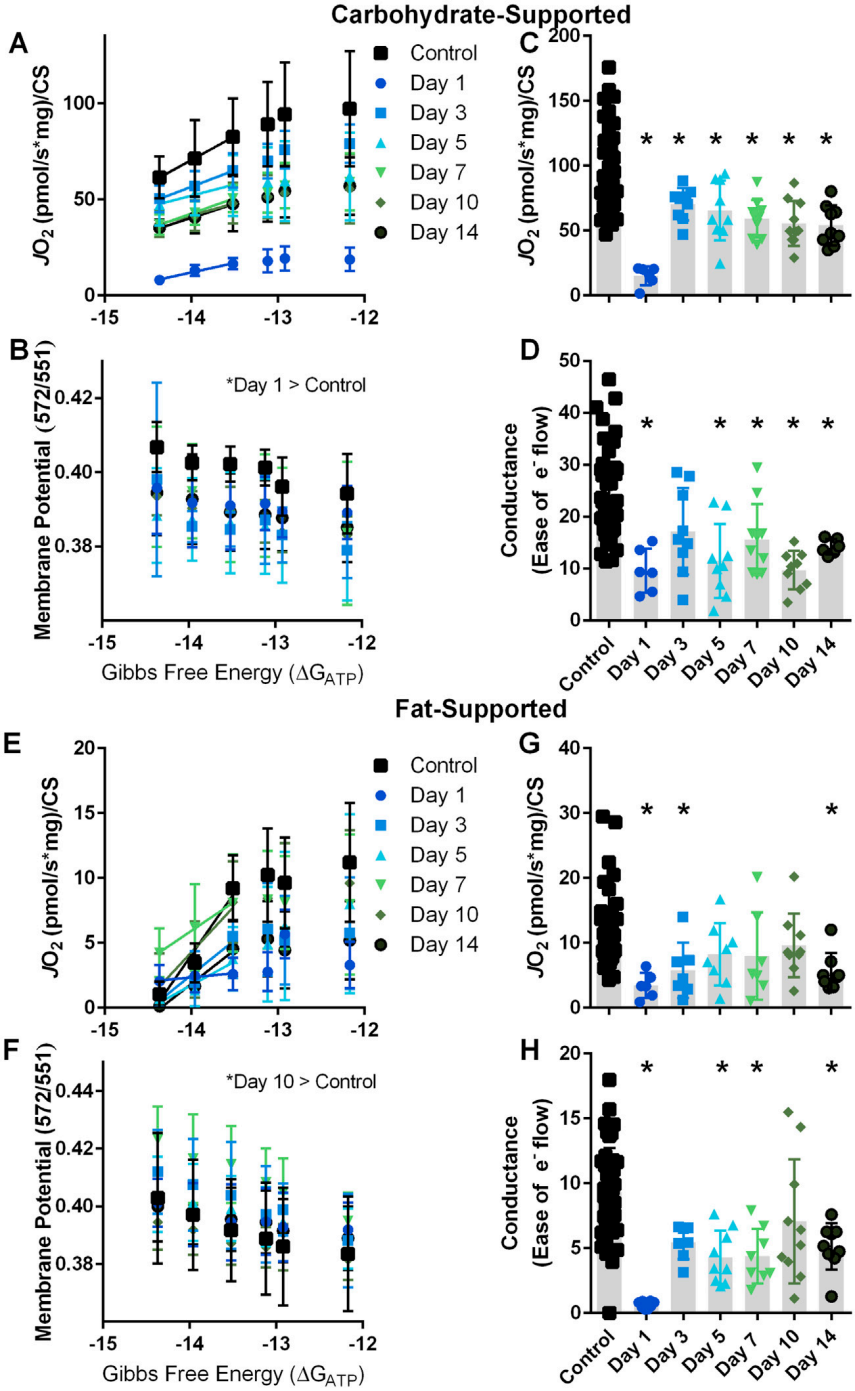
Carbohydrate- and fat-supported respiration and mitochondrial membrane potential at clamped ATP Gibbs free energy states. **(A, B)** Carbohydrate-supported JO_2_ and mitochondrial membrane potential were measured at decreasing ATP re-synthesis demand Gibbs free energy states in uninjured control myofibers and myofibers at 1, 3, 5, 7, 10, and 14 days post-VML injury. **(C)** JO_2_ at −12.17 ∆G_ATP_. **(D)** Electron conductance calculated by the slope of the −14.37, −13.96, and −13.52 ∆G_ATP_ JO_2_ rates in panel **(A)**. **(E, F)** Fat-supported JO_2_ and mitochondrial membrane potential were measured at decreasing ATP re-synthesis demand Gibbs free energy states in uninjured control myofibers and myofibers at 1, 3, 5, 7, 10, and 14 days post-VML injury. **(G)** JO_2_ at −12.17 ∆G_ATP_. **(H)** Electron conductance calculated by the slope of the −14.37, −13.96, and −13.52 ∆G_ATP_ JO_2_ rates in panel **(E)**. Data are shown as mean ± SD. * indicates statistically significant difference compared to uninjured control, *p* < 0.05 analyzed *via* one-way ANOVA.

Independent of injury, JO_2_ diminished with more negative ∆G_ATP_ during the CK Clamp test (−35% with carbohydrate substrates, *p* < 0.001, Figure 2A; and −78% with fat substrates, *p* < 0.001; Figure 2E). This ∆G_ATP –_ *J*O_2_ relationship agrees with the theoretical and experimental relationship reported between ATP free energies and JO_2_ in other tissues (Curtis et al., 2005; U.S. Department of Health and Human Services and U.S. Department of Agriculture, 2015–2020; Southern et al., 2019). In general, a more negative ∆G_ATP_ also produced a greater polarized mitochondrial membrane independent of injury (+5% with carbohydrate substrates, *p* < 0.001, Figure 2B; and +7% with fat substrates, *p* < 0.001; Figure 2F). This *J*O_2_ – mitochondrial membrane potential relationship during the CK Clamp test agrees with similar reports in uninjured skeletal and cardiac muscle, brown adipose tissue, kidney, and liver using this technique (Ahima and Antwi, 2008; Iwasa et al., 2018; Southern et al., 2019), and further support the parallel measurement of these systems to comprehensively elucidate VML-injured myofiber mitochondria function.

### General observation of Carbohydrate- and fat-supported JO_2_ and electron Conductance

In both uninjured and VML-injured myofibers, *J*O_2_ was greater for carbohydrate substrates than fat substrates (Figures 2A, C vs. 2E, G) implying that the oxidative capacity to metabolize pyruvate/malate/succinate is higher compared to palmitoyl-carnitine/malate. Additionally, the linear phase of the CK Clamp relationship between JO_2_ and ∆G_ATP_ has been used previously to define electron conductance (i.e., ease of flow) through the electron transport chain (Glancy et al., 2013; Fisher-Wellman et al., 2018), and reflects mitochondria sensitivity to changing energetic demands. Herein, electron conductance was calculated from the linear portion of the ∆G_ATP_ – *J*O_2_ curve and is represented as the slopes between data points at −14.37 and −13.52 ∆G_ATP_ **(** Figures 2A–E **)**. In agreement with the literature (Fisher-Wellman et al., 2018), myofibers had a greater conductance with carbohydrate substrates compared to fat substrates (Figures 2D vs. 2H).

Recent reports have demonstrated that whole-body metabolism of carbohydrates and fats is influenced by VML injury. Specifically, when 24-h metabolic rate was tracked longitudinally, there was a 10% decrease at 2-week post-injury compared to pre-injury, and the metabolic rate remained attenuated out to 6-week post-injury (Dalske et al., 2021). This same report also assessed the respiratory exchange ratio (RER), a technique that analyzes O_2_ consumption to CO_2_ production to give an indication of carbohydrate and fat metabolism. VML-injured mice had a 4% decrease in RER at 6-week post-injury owing to a decline in carbohydrate metabolism. Whole-body RER estimates of substrate utilization and skeletal muscle-specific substrate-utilization are not the same. However, the latter can influence the former so we decided to interrogate the effect of VML injury on carbohydrate- and fat-supported *J*O_2_ and electron conductance.

### Effect of VML injury on carbohydrate-supported JO_2_, electron conductance, and mitochondria membrane potential.

At the highest demand for ATP re-synthesis, *J*O_2_ was statistically different across the acute time course of VML pathology (Figure 2C). Electron conductance was significantly less at days 1 (−60%), 5 (−52%), 7 (−35%), 10 (−59%) and 14 (−41%) post-VML compared to uninjured control myofibers (Figure 2D). These data support that the VML injury sequela negatively influences the myofiber’s ability to metabolize the carbohydrate substrates at least in the initial 2 weeks following injury, a key time frame for the initiation of muscle regeneration (Quarta et al., 2017; Aguilar et al., 2018; Anderson et al., 2019).

*J*O_2_ and electron conductance are intertwined with mitochondrial membrane potential such that a higher energetic membrane (i.e., more polarized) can apply a greater backpressure on the proton pumping complexes of the electron transport chain and result in a slowing of electron conductance and decrease in *J*O_2_. There was a significant difference in mitochondrial membrane potential (TMRM 572/551 ratio) compared to uninjured controls at day 1 (+5.5%) post-VML injury (*p* < 0.001).

### Effect of VML injury fat-supported JO_2_, electron Conductance, and mitochondrial membrane potential

In contrast to carbohydrate-supported metabolism, there were fewer acute time points with significant differences in *J*O_2_, i.e., days 1 (−70%), 3 (−50%), and 14 (−52%) were significantly different from uninjured control myofibers (*p* = 0.002; Figure 2G); while there were several differences in fat-supported electron conductance (*p* = 0.001) at day 1 (−93%), 5 (−54%), 7 (−53%), and 14 (−45%) compared to uninjured control myofibers (Figure 2H). It is possible fat-supported respiration is more resilient to changes during the acute VML pathology; but it is also possible that fat-respiration rates are too close to the lower detectable range of the device that it obscures the fidelity and interpretation of the results (aka “floor effect”). There was a significant difference in mitochondrial membrane potential (TMRM 572/551 ratio) compared to uninjured controls at day 10 (+8.8%) post-VML injury (*p* < 0.01).

All *J*O_2_ rates reported in Figure 2 are normalized to CS activity. It is notable that there wasn’t a statistical difference in CS activity across the acute time course of VML injury compared to uninjured controls (*p* = 0.257; CS activity avg. 334 ± 58 mmol/min/g) in agreement with a previous report on the early time course of VML injury (Corona et al., 2018).

### The effect of VML injury on the JO_2_ and mitochondrial membrane potential relationship

**FIGURE 3:**
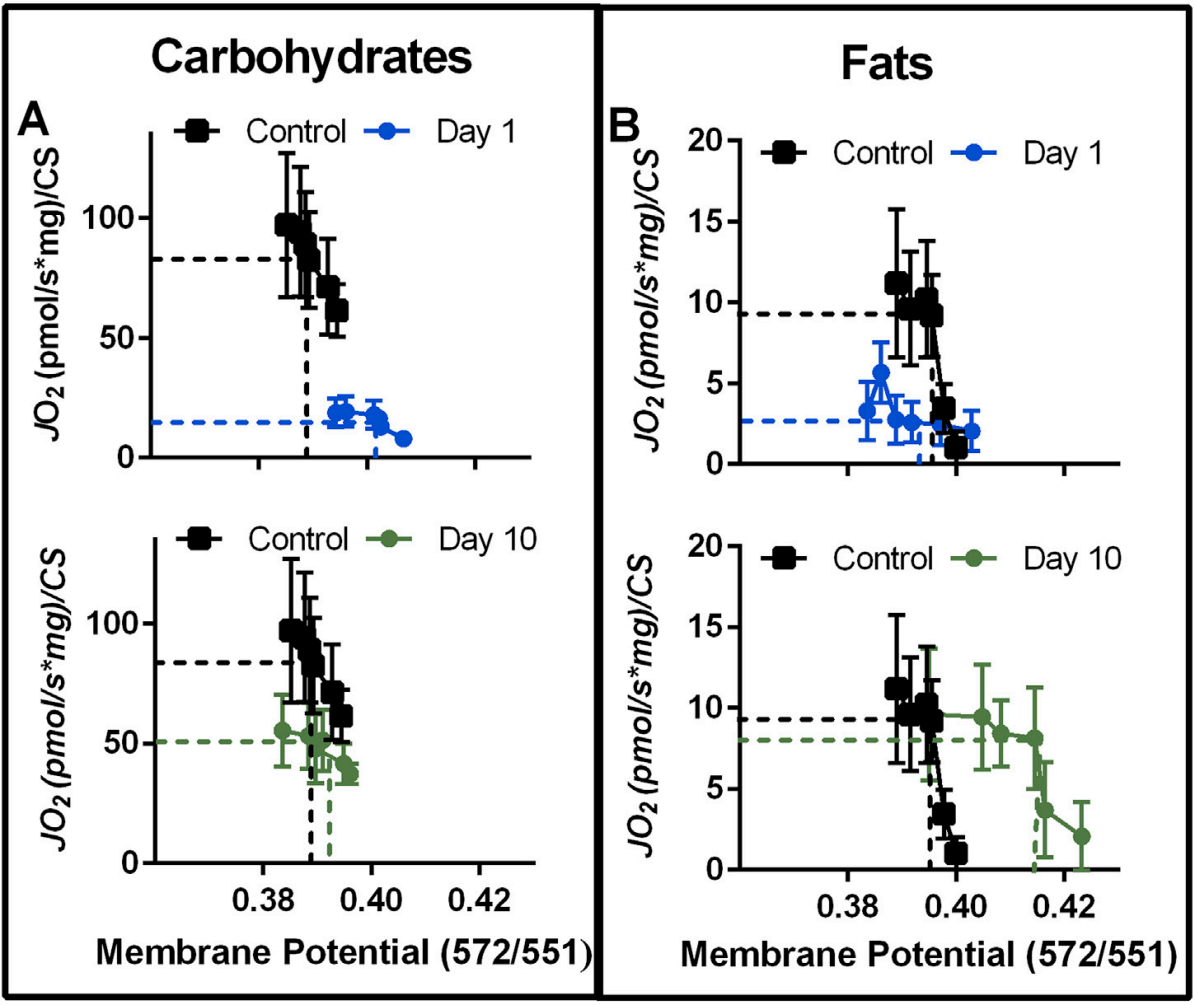
Representative time course of VML effect on the mitochondrial respiration—mitochondrial membrane potential relationship with carbohydrate or fat fuels. **(A, B)** The relationship between mitochondrial membrane potential and JO_2_ with carbohydrate fuels **(A)** and fat fuels **(B)** at 1 and 10 days post-VML injury relative to uninjured control. Data reflects JO_2_ and mitochondrial membrane potentials during the CK Clamp experiment. In both panels, dashed lines highlight JO_2_ and membrane potential relationships at −13.52 ∆G_ATP_ to help visualize shifting inter-relationships. Data are mean ± SD for JO_2_.

We next plotted *J*O_2_ and mitochondrial membrane potential across the entire ∆G_ATP_ curve at two acute timepoints (1- and 10-day post-injury) for carbohydrate and fat substrates (Figure 3). This can be useful to observe the changing relationship between the proton motive force and oxygen consumption during the acute time course of VML injury, and 1- and 10-day post-injury were selected because these timepoints had significant difference in membrane potential, for carbohydrate- and fat-substrates, respectively (Figures 2B, F). For carbohydrate-supported respiration (Figure 3A), the effect of the VML injury is a downward-rightward shift such that for any ∆G_ATP_ tested, VML-injured myofibers had less *J*O_2_ and a greater polarized mitochondrial membrane at Day 1. The membrane potential component of this relationship was not present at Day 10.

In contrast, fat-supported plots (Figure 3B) demonstrate a downward shift (driven by less *J*O_2_ for any given ∆G_ATP_) at Day 1 and a rightward shift (driven by greater membrane polarization) at Day 10. A greater energetic potential at the mitochondrial membrane in concert with less *J*O_2_ could indicate a higher resistance to ATP synthesis. However, the mitochondrial network becomes highly fractured and disorganized during this time after VML injury (Southern et al., 2019; Forouhesh Tehrani et al., 2021) and one of the determinants of mitochondrial function is its location and structural organization (Ferreira et al., 2010; Glancy et al., 2015; Glancy et al., 2017). The mitochondrial network connectively has been shown to rapidly distribute changes in mitochondrial membrane potential (Glancy et al., 2015; Glancy et al., 2017), effectively distributing energy more rapidly in a cell than would otherwise be possible *via* substrate diffusion alone. Furthermore, the mitochondrial network can rapidly sequester damaged mitochondrial units to halt the spreading of damage to the network. Therefore, the relationship between *J*O_2_ and mitochondrial membrane potential is likely more complex than just a higher resistance to ATP synthesis.

## Conclusion

The use of mitochondrial respiration techniques in general to evaluate the severity of muscle damage is a relatively unused approach in the field of VML. However, cataloguing the decline in mitochondrial metabolism after severe injury may prove important to describing whole-body metabolic changes after injury as well as identifying new therapeutic targets to improve the overall function of the remaining muscle after a VML injury.

Our results validate the CK Clamp approach as a useful tool in identifying a dynamic relationship in mitochondrial respiration and mitochondrial membrane potential for carbohydrate substrates during acute VML. Despite previous studies evaluating mitochondrial respiration using methods that don’t recapitulate *in vivo* physiological conditions, the findings reported in this brief report support their conclusion that VML injury causes damage to the metabolic function of the remaining myofibers. Several bioenergetic questions remain for the short- and long-term VML pathology; including, how the ATP/ADP and NAD(P)/NAD(P)H ratios are affected by the injury, specific electron transport chain complex sensitivity to the VML injury, reactive oxygen species production, and importantly, what is the initial mechanism of the metabolic pathology (e.g., loss of calcium homeostasis and/or mitochondria network disorganization). The CK Clamp technique is a valuable tool in pursuit of answering these questions following VML injury.

## Data Availability

The original contributions presented in the study are included in the article/supplementary material, further inquiries can be directed to the corresponding author.
